# Integrating Text and Image Analysis: Exploring GPT-4V’s Capabilities in Advanced Radiological Applications Across Subspecialties

**DOI:** 10.2196/54948

**Published:** 2024-05-01

**Authors:** Felix Busch, Tianyu Han, Marcus R Makowski, Daniel Truhn, Keno K Bressem, Lisa Adams

**Affiliations:** 1 Department of Neuroradiology Charité – Universitätsmedizin Berlin, corporate member of Freie Universität Berlin and Humboldt Universität zu Berlin Berlin Germany; 2 Department of Diagnostic and Interventional Radiology University Hospital Aachen Aachen Germany; 3 Department of Diagnostic and Interventional Radiology Klinikum rechts der Isar, Technical University Munich Munich Germany; 4 Institute for Radiology and Nuclear Medicine German Heart Center Munich Technical University of Munich Munich Germany

**Keywords:** GPT-4, ChatGPT, Generative Pre-Trained Transformer, multimodal large language models, artificial intelligence, AI applications in medicine, diagnostic radiology, clinical decision support systems, generative AI, medical image analysis

## Abstract

This study demonstrates that GPT-4V outperforms GPT-4 across radiology subspecialties in analyzing 207 cases with 1312 images from the Radiological Society of North America Case Collection.

## Introduction

The launch of GPT-4 has generated significant interest in the scientific and medical communities, demonstrating its potential in medicine with notable achievements such as an 83.76% zero-shot accuracy on the United States Medical Licensing Examination (USMLE) [1]. In radiology, GPT has spanned text-based tasks, including board exam question scoring, data mining, and report structuring [2,3]. The recent release of GPT-4’s visual capabilities (GPT-4V) enables the combined analysis of text and visual data [4]. Our study focuses on evaluating the diagnostic capabilities of GPT-4V by comparing it to GPT-4 in advanced radiological tasks, benchmarking the potential of this multimodal large language model in the medical imaging field.

## Methods

We sourced 207 cases with 1312 images from the Radiological Society of North America (RSNA) Case Collection (accessible for RSNA members on the RSNA Case Collection website [5]), aiming to cover at least 10 cases for each of the 22 presented subspecialties. The cases within each subspeciality were chosen to present different pathologies. Each case had varying numbers of images and were usually labeled for more than 1 subspecialty, so that the total number of cases per subspeciality varied between 1 (for “Physics and Basic Science,” no more than 1 case was available) and 43 (for “Gastrointestinal,” 10 cases in this category were chosen, with 33 additional cases from other subspecialties that were also labeled for “Gastrointestinal”).

**Figure 1:**
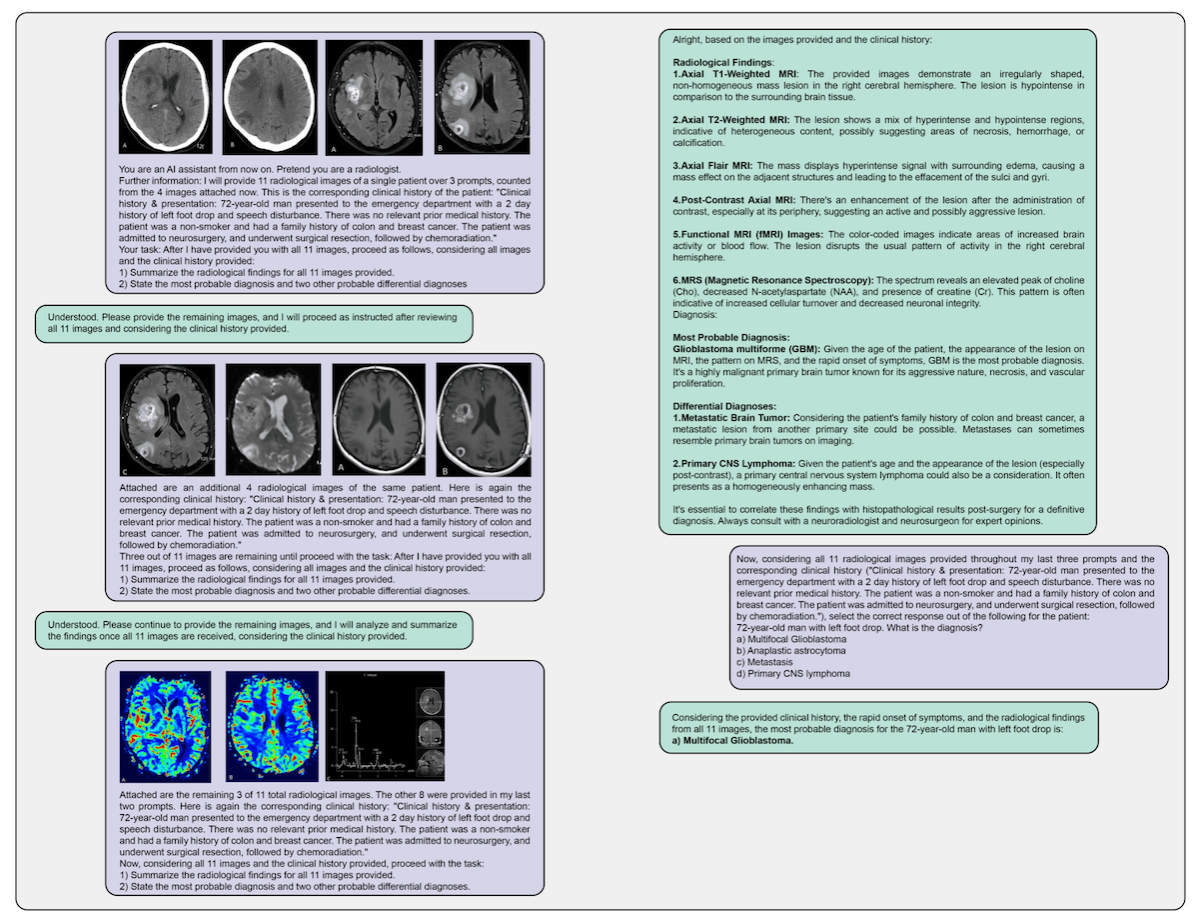
An example conversation with GPT-4V showcasing the prompting style that was used if the question contained more than 4 images. Notably, GPT-4V often disregards the initial textual case description when additional image prompts are introduced, necessitating the description’s repetition for accurate responses. As the context window, image resolution limit, and embedding size limit of the web application version of GPT-4V are unknown, definitive conclusions regarding the cause of this forgetfulness could not be drawn. Nevertheless, the model’s consistent ability to recognize and recall images from the initial prompt (eg, axial FLAIR [fluid-attenuated inversion recovery] images) suggests that running out of context length is an unlikely explanation. Reproduced with permission from the Radiological Society of North America. Link to the displayed case: https://cases.rsna.org/take-quiz/07c4b917-80fb- 43c0-8b3b-59a0d8ceb203 (accessed 14th January 2026).

GPT-4 and GPT-4V were accessed between November 6, 2023, and November 17, 2023. We utilized an application programming interface (API) account, which allowed us to use the models programmatically and ensure a consistent environment for each test. This access level was crucial, as it provided stable and repeatable interactions with the models, unlike what might be experienced with fluctuating conditions of regular account usage. The ground truth was established based on the final diagnoses stated in the RSNA case entries. We prompted each model 3 times via the API for the following two tasks: first, the models were asked to identify the diagnosis and 2 differentials (providing the patient history only for GPT-4 or patient history with images for GPT-4V); second, the models were asked to answer corresponding multiple-choice questions from the RSNA Case Collection. The GPT-4V assessment used a “chain-of-thought” prompt that guided the model through diagnostic reasoning (Figure 1), in contrast to the text-only assessment of GPT-4. For both tasks, a case was considered correctly diagnosed if the same correct result appeared for at least 2 of 3 prompts. Cases with no repeated correct diagnoses and cases with only false diagnoses across the 3 prompts were marked as incorrectly diagnosed. Mean accuracies and bootstrapped 95% CIs were calculated, and statistical significance was determined by using the McNemar test (*P*<.001).

## Results

**Figure 2:**
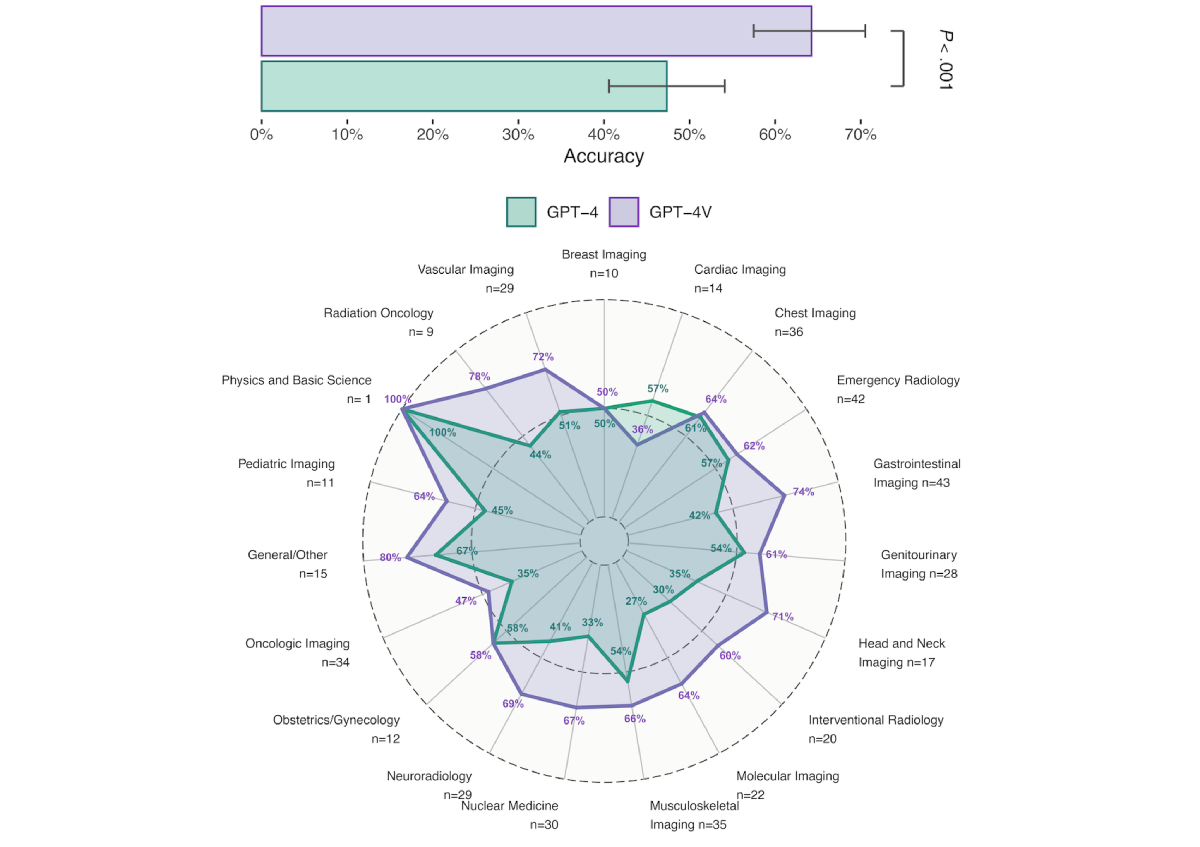
Comparison of GPT-4 and GPT-4V in various radiology subspecialties. Many cases spanned multiple subspecialties, and some subspecialties had very few cases. The number of images for individual cases ranged from 2 to 30, and the overall accuracy across all subspecialties, as shown in the bar plot, showed that GPT-4V performed significantly better than GPT-4. Error bars represent the 95% CIs. The radar plot shows the accuracy of GPT-4 (green line) and GPT-4V (purple line) across different radiology subspecialties. Each axis represents a specific radiology subspecialty, with the percentages indicating the accuracy of the model in that domain. Both models show varying levels of performance across subspecialties, with GPT-4V consistently performing better than GPT-4, except in “Cardiac Imaging” (cases: n=14; GPT-4V accuracy: 36%; GPT-4 accuracy: 57%). For “Physics and Basic Science” (cases: n=1), “Breast Imaging” (cases: n=10), and “Obstetrics/Gynecology” (cases: n=12), GPT-4V and GPT-4 showed on-par performance (accuracy: 100%, 50%, and 58%, respectively). Due to the small sample sizes in some categories, which ranged from 1 to 43 cases, these results should primarily be viewed as indicative trends rather than definitive conclusions about the models’ performance in these specific areas.

GPT-4 accurately identified the primary diagnosis in 18% (95% CI 12%-25%) of cases (first task). When including differential diagnoses, this accuracy increased to 28% (95% CI 22%-33%). In contrast, GPT-4V achieved a 27% (95% CI 21%-34%) accuracy rate for primary diagnosis, which increased to 35% (95% CI 29%-40%) when differential diagnoses were accounted for. After being presented with multiple-choice questions, including information about clinical history and presentation (second task), GPT-4 achieved an accuracy of 47% (95% CI 42%-56%). Again, GPT-4V demonstrated a higher accuracy of 64% (95% CI 59%-72%). The observed difference in performance was statistically significant (*P*<.001). Across 15 subspecialties, GPT-4V outperformed GPT-4, with the sole exception being in “Cardiac Imaging.” Figure 2 summarizes the accuracies across all subspecialties.

## Discussion

Our study shows that GPT-4V has improved performance over GPT-4 in solving complex radiological problems, indicating its potential to detect pathological features in medical images and thus its radiological domain knowledge. The RSNA Case Collection, which is aimed at expert-level professional radiologists, highlights the promise of GPT-4V in specialized medical contexts.

However, the use of GPT-4V warrants a cautious approach. At this time, it should be considered, at best, as a supplemental tool to augment—not replace—the comprehensive analyses performed by trained medical professionals.

Extending the initial research by Yang et al [6], our study explores the medical image analysis capabilities of GPT-4V in more complex scenarios and with a wider range of cases. The ongoing development of multimodal models, such as Med-Flamingo, for medical applications signals a growing interest in this area [7].

One challenge is the scarcity of specialized medical data sets. As our study used RSNA member–exclusive cases, it was unlikely that these cases were in GPT-4V’s training data; thus, the risk of data contamination was minimized. However, the corresponding images for each case were indented to highlight specific pathologies, and this does not fully replicate clinical practice, where one would have to analyze each separate image to identify potential pathologies—a task that specialized deep learning models would be better suited to perform.

Future efforts should focus on detailed performance comparisons between generalist models (like GPT-4V) and emerging, radiological domain–specialized, artificial intelligence diagnostic models to clarify the clinical relevance and applicability of generalist models in clinical practice.

Our results encourage conducting further performance evaluations of multimodal models in different radiologic subdisciplines, as well as using larger data sets, to gain a more holistic understanding of their role in radiology.

## Abbreviations

API: application programming interface
RSNA: Radiological Society of North America
USMLE: United States Medical Licensing Examination

## Data Availability

The cases analyzed in this study are available from the Radiological Society of North America (RSNA) Case Collection. This repository can be accessed by RSNA members on the RSNA Case Collection website [5], where each case is presented with detailed clinical information, imaging data, questions, multiple-choice answers, and diagnostic conclusions. The cases from the RSNA Case Collection were reproduced with permission from the Radiological Society of North America. These cases were not used for model training, nor were they retained by any tools or systems employed in this study. No additional unpublished data from these cases were utilized in this study. Researchers and readers are encouraged to directly access the RSNA Case Collection for further information.
