# Correction: Integrating Text and Image Analysis: Exploring GPT-4V’s Capabilities in Advanced Radiological Applications Across Subspecialties

**DOI:** 10.2196/64411

**Published:** 2024-07-19

**Authors:** Felix Busch, Tianyu Han, Marcus R Makowski, Daniel Truhn, Keno K Bressem, Lisa Adams

**Affiliations:** 1 Department of Neuroradiology Charité – Universitätsmedizin Berlin, corporate member of Freie Universität Berlin and Humboldt Universität zu Berlin Berlin Germany; 2 Department of Diagnostic and Interventional Radiology University Hospital Aachen Aachen Germany; 3 Department of Diagnostic and Interventional Radiology Klinikum rechts der Isar, Technical University Munich Munich Germany; 4 Institute for Radiology and Nuclear Medicine German Heart Center Munich Technical University of Munich Munich Germany

In “Integrating Text and Image Analysis: Exploring GPT-4V’s Capabilities in Advanced Radiological Applications Across Subspecialties” (J Med Internet Res 2024;26:e54948) one error was noted.

In the second paragraph of the “Methods” section, the phrase:

GPT-4 and GPT-4V were accessed between November 6, 2023, and November 17, 2024.

Has been changed to read as follows:

GPT-4 and GPT-4V were accessed between November 6, 2023, and November 17, 2023.

The correction will appear in the online version of the paper on the JMIR Publications website on July 19, 2024, together with the publication of this correction notice. Because this was made after submission to PubMed, PubMed Central, and other full-text repositories, the corrected article has also been resubmitted to those repositories.

