# Correction: Integrating Text and Image Analysis: Exploring GPT-4V’s Capabilities in Advanced Radiological Applications Across Subspecialties

**DOI:** 10.2196/91415

**Published:** 2026-02-11

**Authors:** Felix Busch, Tianyu Han, Marcus R Makowski, Daniel Truhn, Keno K Bressem, Lisa Adams

**Affiliations:** 1 Department of Neuroradiology Charité – Universitätsmedizin Berlin, corporate member of Freie Universität Berlin and Humboldt Universität zu Berlin Berlin Germany; 2 Department of Diagnostic and Interventional Radiology University Hospital Aachen Aachen Germany; 3 Department of Diagnostic and Interventional Radiology Klinikum rechts der Isar, Technical University of Munich Munich Germany; 4 Institute for Radiology and Nuclear Medicine German Heart Center Munich Technical University of Munich Munich Germany

In “Integrating Text and Image Analysis: Exploring GPT-4V’s Capabilities in Advanced Radiological Applications Across Subspecialties” [1], the authors made two corrections.

The following text was added to the caption of Figure 1:

Reproduced with permission from the Radiological Society of North America. Link to the displayed case: https://cases.rsna.org/take-quiz/07c4b917-80fb- 43c0-8b3b-59a0d8ceb203 (accessed 14th January 2026).

In the Data Availability section, the following sentence was revised:

The data set was utilized under the terms and conditions provided by the RSNA, which permits the use of these cases for educational and research purposes.

This section now reads:

The cases from the RSNA Case Collection were reproduced with permission from the Radiological Society of North America. These cases were not used for model training, nor were they retained by any tools or systems employed in this study.

The correction will appear in the online version of the paper on the JMIR Publications website, together with the publication of this correction notice. Because this was made after submission to PubMed, PubMed Central, and other full-text repositories, the corrected article has also been resubmitted to those repositories.
